# Modeling Diet-Induced Obesity with Obesity-Prone Rats: Implications for Studies in Females

**DOI:** 10.3389/fnut.2016.00050

**Published:** 2016-11-24

**Authors:** Erin D. Giles, Matthew R. Jackman, Paul S. MacLean

**Affiliations:** ^1^Department of Nutrition and Food Science, Texas A&M University, College Station, TX, USA; ^2^Anschutz Health and Wellness Center, University of Colorado Anschutz Medical Campus, Aurora, CO, USA; ^3^Division of Endocrinology, Diabetes and Metabolism, Department of Medicine, University of Colorado Anschutz Medical Campus, Aurora, CO, USA

**Keywords:** obesity resistance, weight regain, menopause, breast cancer, exercise, adipose, high-fat diet, sex differences

## Abstract

Obesity is a worldwide epidemic, and the comorbidities associated with obesity are numerous. Over the last two decades, we and others have employed an outbred rat model to study the development and persistence of obesity, as well as the metabolic complications that accompany excess weight. In this review, we summarize the strengths and limitations of this model and how it has been applied to further our understanding of human physiology in the context of weight loss and weight regain. We also discuss how the approach has been adapted over time for studies in females and female-specific physiological conditions, such as menopause and breast cancer. As excess weight and the accompanying metabolic complications have become common place in our society, we expect that this model will continue to provide a valuable translational tool to establish physiologically relevant connections to the basic science studies of obesity and body weight regulation.

## Introduction

Worldwide obesity rates have more than doubled since the 1980s, and in 2014 more than 39% of adults were overweight, and 13% were obese (1). This translates to an estimated 1.9 billion adults who are overweight, of whom more than 600 million are obese. Further, 41 million children under the age of 5 are also overweight or obese, making obesity a worldwide epidemic. While obesity was once only a problem for high-income countries, there are now more deaths worldwide attributed to overweight and obesity than underweight. Obesity negativity affects virtually every system of the body and increases the risk for cardiovascular disease, diabetes, osteoarthritis, and many cancers. While childhood obesity increases the risk of obesity in adulthood, it also poses immediate risks to children, such as breathing difficulties, increased risk of fractures, and psychological effects. Further, markers of metabolic disease that were once considered limited to adults are now appearing in children with obesity, including hypertension, insulin resistance, and early markers of cardiovascular disease. Clearly our current efforts to stop this growing epidemic are not working, and there is an urgent need to understand the etiology and to develop new strategies, interventions, and therapies to prevent and/or treat this disease.

It is generally accepted that the problem of obesity reflects the merger of environmental, biological, and psychosocial pressures (2). Underlying the biological component of this issue is the genetic and epigenetic foundation that establishes the systems controlling energy homeostasis and body weight regulation. The heterogeneity of this foundation imparts variability in how these systems function and respond to external challenges and stressors. Even in the current obesogenic environment, not all individuals become obese. While some individuals have a predisposition to accumulate excess weight, others have a predisposition to remain lean. For the vast majority of the population, the variability in the predisposition for obesity is not linked to a single mutation or epigenetic insult. Rather, numerous genes and epigenetic events are involved in generating a polygenic predisposition that favors leanness, obesity, or some level of adiposity between the two extremes, when faced with obesogenic environmental pressures.

One environmental pressure believed to be promoting obesity is the availability of energy dense diets. In animal models, this has been shown to increase energy intake (3–6) and eventually leads to the development of obesity and insulin resistance (7–10). However, the susceptibility to obesity in response to this challenge is highly dependent upon the strain of the animal (11–18). The purpose of this review is to summarize the development, nuances, and applications of an approach that models this polygenic susceptibility to the development of obesity in response to the common environmental pressure of a freely available energy dense diet. This experimental paradigm has been, and continues to be, a valuable tool to translate the wealth of knowledge from basic science studies of energy balance to physiological relevant aspects of the human condition.

## Diet-Induced Obesity (DIO) from Obesity-Prone Animals

### DIO in the Context of Alternative Models of Obesity

Numerous types of animal models have been employed to study obesity. Certain mutations, as with leptin or the leptin receptor, give rise to monogenic forms of obesity with extreme phenotypes (19). These models have proven very valuable in elucidating the function of specific factors involved in energy homeostasis and body weight regulation. It has become clear, however, that the biological contribution to obesity in humans involves numerous factors in a number of tissues that coordinately favor the accumulation and maintenance of an excess amount of adiposity. Translating these observations to the human condition requires applying this information in a more physiologically relevant context.

As an alternative, different strains of animals with a known genetic disposition for leanness or obesity have been studied. The Osborne–Mendel (OM) and S5B/P1 model is one such example. In studies comparing several strains of rats on a high fat (HF) diet, OM rats were identified as prone to the development of obesity, while S5B/P1 rats were resistant (8). Using such models has helped researchers identify genetic differences that contribute to the predisposition to develop obesity. However, the heterogeneity between these two strains also imposes unwanted variability in comparative studies that may be irrelevant or confounding.

To overcome the potential confound of strain differences, we and others have utilized an approach that yields a range of adiposity phenotypes within the same strain. Because a diet high in fat has been linked to obesity in both humans and animal models, this has become the most common challenge used to select the extremes of adiposity phenotypes. The strengths of this approach are that the polygenic predisposition for obesity reflects a differential response to the diet, while minimizing the extraneous differences between strains. This general approach has been used with both inbred and outbred strains of rats and mice to select those that become lean or obese with the same dietary challenge. Selection from inbred strains, which are considered to be isogenic, provides a more modest range of phenotypes that presupposes less genetic variability and emphasizes epigenetic variability in driving the phenotype. In contrast, selection from outbred strains presupposes greater variability in both genetic and epigenetic differences, which likely better reflects the susceptibility to obesity in humans. The diet-induced obesity (DIO)/diet-resistant (DR) model of obesity that we emphasize in the discussion of this review are derived from outbred rats (15, 18).

Over the past two decades, some confusion has emerged in the terminology used in describing outbred models. We and others have often referred to them as obesity-prone (OP) and obesity-resistant (OR), rather than DIO and DR. The two different naming schemes have often been used interchangeably, while others have inferred some distinction between them. Here, we propose that both classifications have value and are inherently related, in that the polygenic predisposition (OP/OR) ultimately leads to the respective phenotype (DIO/DR), when challenged with the obesogenic selection diet. To minimize the confusion in future studies, we propose the following definitions that distinguish the use of these terms:

• OP: a person or animal that has a predisposition to become obese when challenged with an obesogenic environmental pressure;
• OR: a person or animal that has a predisposition to remain lean when challenged with the same obesogenic environment;
• DIO: the OP person or animal that has become obese in response to the obesogenic diet; and
• DR: the OR person or animal that has remained lean in response to the obesogenic diet.

### Evolution of the Selection Process – A Historical Perspective

Selection and study of OP/OR phenotypes began over 25 years ago in both Sprague-Dawley and Wistar outbred rats (18, 20). In general, a relatively large group of outbred rats was challenged with a HF diet for a defined period of time, and the cohort was stratified by the amount of weight gained. For the early studies from Hill et al. (21, 22), Wistar rats were obtained from Harlan Laboratories (Madison, WI, USA; now Envigo). The low fat (LF) acclimation diet consisted of a 20% LF diet (20% kcal from fat; 20% protein; 60% carbohydrate) for 2 weeks. A 60% HF diet (60% kcal from fat; 20% protein; 20% carbohydrate) was then fed for 4–5 weeks, and the top and bottom 25% of weight gainers were identified as either OP or OR. At the end of the 10-week study, body composition and fat pad weights confirmed the DIO and DR phenotypes. Subsequent modifications to the protocol selected the top and bottom tertiles (rather than quartiles) to represent the extremes of weight gain (23). During a similar time period, Levin published similar findings using a different strain and a different obesity-inducing diet. In these studies, male Sprague-Dawley rats were fed a diet consisting of chow, corn oil, and sweetened condensed milk [~31% kcal fat and 45–53% kcal carbohydrate (primarily sucrose)]. Like the Wistar rats, only about half of the rats consuming this diet for 3–5 months gained excess weight compared to chow-fed controls (20, 24–26).

Over the years, there have been a number of different modifications to the design of the selection diet that are particularly relevant to note. Levin’s group has consistently employed a diet that contains sucrose, in addition to being high in fat. In some studies, Levin’s group also used the highly palatable liquid diet Ensure (27, 28). In contrast, Hill’s research team moved to LF and HF diets with 12 and 45% kcal from fat, with the protein component held constant at 20%. Further, the sucrose component of the original HF diet was replaced with starch (29). Finally, in conjunction with a move to the University of Colorado in 1997, Charles River Laboratories (Wilmington, MA, USA) became the source for Wistar rats, while maintaining the 12 and 45% fat for the LF and HF diets, respectively (14).

In 1994, Pagliassotti et al. modified the selection protocol to demonstrate that weight gain during the first week on the HF diet was highly predictive of weight gain over the four subsequent weeks. Specifically, they reported a strong correlation between weight gain after 1 and 5 weeks of HF feeding (*r* = 0.87; *n* = 200) (12). Based on this finding, most subsequent studies have relied on this shorter duration of HF diet screening for identifying OP and OR rats. In collaboration with Hill’s group, a study out of the Leibowitz lab (30) used a similar model in Sprague-Dawley rats to identify measures in prepubertal animals that were predictive of adult adiposity. Similar to studies in Wistar rats from the Hill lab, they found that weight gain across a 5-day interval from 30 to 35 days of age on a HF diet (45–60% kcal fat) was strongly and positively correlated (*r*^2^ = 0.71–0.82) with accumulated body fat in four depots after 4–6 weeks of HF feeding. Screening in younger animals did not show this same correlation, suggesting that waiting until 4–5 weeks of age is necessary to identify OP and OR phenotypes using this model (30). Similarly, Levin’s group have also shown that the DIO and DR phenotypes are not different between 3 and 5 weeks of age (31).

Overall, the diet and the timeframe of selection has evolved and varied between groups over the past 25 years. A reduced time frame for selection allows for animals to be studied earlier in the development of the DIO/DR phenotypes. Diet composition has evolved to better reflect a more reasonable and relevant amount of fat (40–50% kcal), and either to include or not include sucrose as a portion of the carbohydrate component. It should be noted that the addition of sucrose can lead to more severe metabolic derangements in the DIO animals that are generated, even to the extent of developing diabetes in some cases.

### Levin’s DIO/DR Model – Selective Inbreeding

One limitation of a model that requires stratification based on weight gain is that differences between OP and OR phenotypes cannot be studied prior to the dietary challenge. To circumvent this limitation, Levin et al. developed inbred lines of DIO and DR rats that were derived from OP and OR Sprague-Dawley rats. Briefly, outbred Sprague-Dawley rats that were either susceptible or resistant to weight gain after 2 weeks on a HF, high energy diet were identified and inbred (15). After five generations of selective inbreeding, the resultant lines breed true to their respective phenotypes, with a bimodal distribution of weight gain in response to the high energy diet. The resulting lines of animals were termed DIO or DR for those that were either susceptible or resistant to DIO, respectively. These valuable lines have provided the means to examine preexisting differences between OP and OR animals, as well as the differential responses that occur within the first few days of exposure to a HF and/or high energy diet.

### Current Selection Protocol for DIO/DR Studies of Obesity

**Figure 1:**
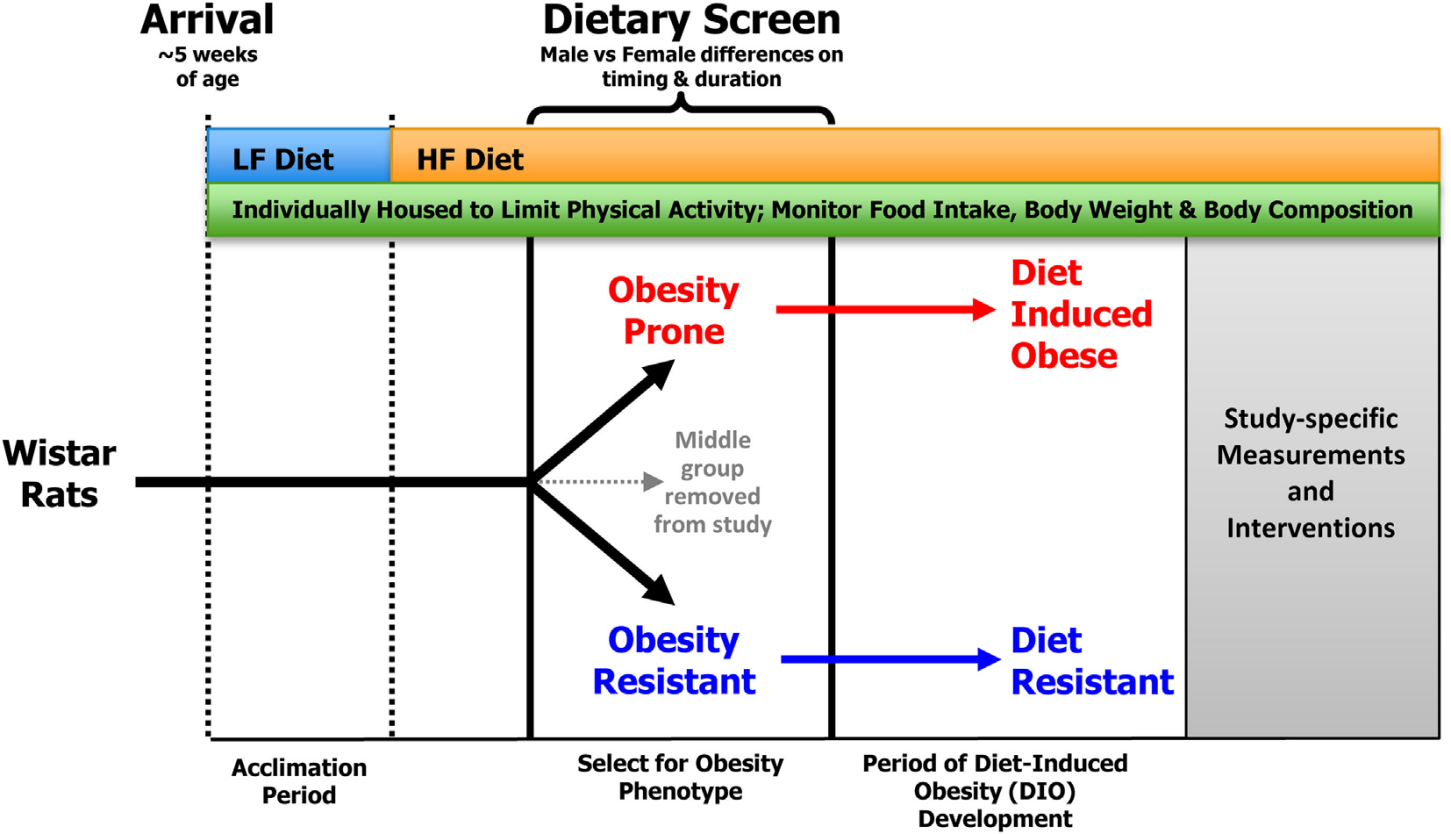
**General screening procedure for selecting OP and OR rats**. Outbred Wistar rats that arrive at ~5 weeks of age are placed in individual wire bottom cages that limit physical activity and allow for accurate measurement of food intake and spill. Food intake, body weight, and body composition are monitored for the duration of the study. Following a brief acclimation period, rats are provided *ad libitum* access to the HF diet, which results in a heterogeneous distribution of body weight gain in the outbred animals. Obesity-prone (OP) and -resistant (OR) animals are identified as described in the text and matured into diet-induced obese (DIO) and diet-resistant (DR) animals, respectively.

In recent years, we have employed a standardized dietary screen of outbred rats (Figure 1) to identify those rats that have a polygenic predisposition for resistance or propensity to become obese under environmental pressures that are thought to contribute to obesity in humans: consumption of a HF diet and limited physical activity (18, 32–35). While we typically use Wistar rats, outbred Sprague-Dawley rats have also been used with success. A number of vendors are available that commercially produce semi-purified diets, but we have typically used Research Diets D12344 and D11724 as our HF (46%) and LF (12%) diets, respectively.

As a general protocol, rats arrive at our facility at ~5 weeks of age. They are individually housed in wire bottom cages and provided free access to a LF diet for an acclimation period of ~2 weeks. This allows time for any institution-required quarantine, recovery from the stress of transport, and an opportunity to perform any baseline measurements. Following acclimation, rats are provided *ad libitum* access to the HF diet, which results in a heterogeneous distribution of body weight gain in the outbred animals. Rats have traditionally been ranked according to their change in body weight in response to HF feeding; those with the lowest weight gain are classified as OR, whereas those with the greatest weight gain are classified as OP. At this point, animals in the middle group are removed from the study. The HF diet is a critical component for the separation of rats into the OP and OR phenotypes as there is no difference in body weight gain when the animals are consuming an LF diet.

In addition to feeding a HF diet, another key requirement for emergence of the OP and OR phenotypes in this model is individual housing in wire bottom cages. Individual housing serves several purposes. First, it limits the physical activity of the animals compared to animals housed in a group environment (36), as group-housed animals are constantly engaged with one another. Second, it allows for more precise measures of food intake, which is often an outcome in studies of this nature. Third, it prevents dominant animals from influencing food intake patterns of other rats in the group (37). Finally, it prevents coprophagia that can be common in studies of energy restriction and weight loss. In most of our studies, we use rat cages measuring ~9.25″(L) × 6.75″(W) × 6.75″(H), although we have also used slightly larger cages with success.

### Developing the DIO/DR Model for Studies in Females

Sex-specific effects and sex differences in outcomes have received greater attention over the last decade as the scientific community has developed an appreciation for the importance of the biological variable of sex. Barry Levin’s inbred DIO/DR lines have been valuable in this regard, as females could be derived directly from the respective inbred lines. The use of outbred strains presented a challenge with the selection of OP and OR rats, in that the unique aspects of female physiology and the associated variability confounded the screening process. Even so, the expense of maintaining Levin’s inbred lines and the tenuous nature of their commercial availability led us to develop a more consistent screening protocol for the more readily available outbred strains. Our initial goal with this work was to combine the OR/OP model with established approaches for studying the loss of ovarian function and mammary tumors to develop paradigm for examining the impact of obesity on postmenopausal breast cancer. In addition to our intended use, this model has applicability to the study of numerous obesity-associated comorbidities in females.

We used the same source for female Wistar rats as we had for male rats in our previous studies: Charles River Laboratories (Wilmington, MA, USA). Identical to our male protocol, the female rats were individually housed in metabolic caging designed to allow for monitoring food intake while minimizing physical activity. Rats were fed the same HF purified diet (46% kcal fat; Research Diets, New Brunswick, NJ, USA; RD#12344) with free access to water.

In early studies with females, Hill’s group successfully separated female rats into OP and OR after 4 weeks on a 60% fat diet containing sucrose (18). In more recent studies, we have observed that separating females into their respective phenotypes is more complicated when the sucrose-free, 46% fat diet is used. In our group’s first published study in females (38), we stratified the rats into tertiles using the male-specific protocol based on change in body weight in response to HF feeding early in life. However, we found body composition in the mature females to be more variable than in the males, such that larger animal could have a lean phenotype and smaller animals could present with a higher level of adiposity. This necessitated the use of a retrospective analysis to identify rats as lean, mid-weight, and obese based on their weight gain from ~8 to 19 weeks of age. A 19-week time point was chosen because this was the time at which weight gain began to taper off, which we interpreted to indicate that the animals were fully mature and likely no longer depositing lean body mass.

In two subsequent studies with females (39, 40), we adjusted our approach and ranked animals by their rate of weight gain in the obesogenic environment from 10 to 18 weeks of age. Using this criteria resulted in a clean separation into obese and lean phenotypes in the mature animals (studied between 20 and 30 weeks of age), in which obese rats had significantly higher body weight, body fat, and circulating triglycerides when compared to their lean counterparts.

Although our 8-week screening protocol generated a clean separation of the OP and OR phenotypes, it was far more labor intensive and costly than the 1-week protocol used in males, where the middle tertile of animals could be transferred out of the study early in life, rather than at 4–5 months of age. Thus, as part of a recent large obesity/menopause/breast cancer study with >300 female rats spread over three cohorts (currently unpublished), we measured body weight and composition at several time points to determine the earliest and shortest period of time that could be used to predict the OP and OR phenotypes in females with an acceptable level of accuracy. Female Wistar rats were individually housed and fed our standard HF diet (46% fat) for the duration of the study. Body weights were measured weekly for the duration of the study, and body composition measurements (qMR, EchoMRI, Houston, TX, USA) were performed at 9, 14, and 18 weeks of age. Body composition was also measured in all animals immediately prior to undergoing surgical ovariectomy (OVX). This occurred at an average of 26.5 ± 0.6 weeks of age and was therefore used as a measure of adult adiposity for the analysis.

**Figure 2:**
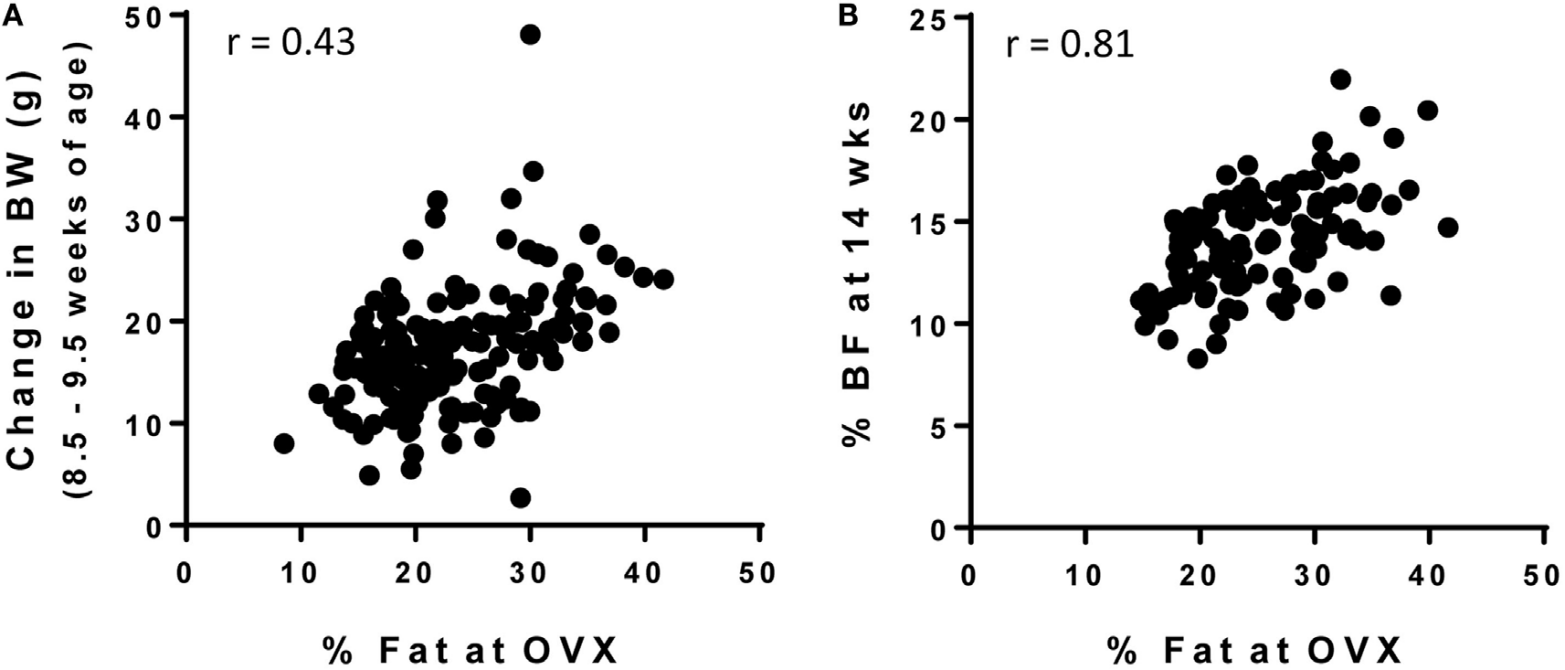
**Screening strategies for OP and OR phenotype in female Wistar rats**. Female rats were individually housed in wire bottom cages and fed a HF diet (46% kcal fat) for the duration of the study. Body weight and body composition were measured throughout the study and was correlated with % body fat at the time of OVX (maturity; 26.5 ± 0.6 weeks of age). **(A)** Change in body weight early in life (8.5–9.5 weeks of age) is not a strong predictor of adult adiposity, but **(B)** % body fat at 14 weeks of age does predict adult adiposity.

As shown in Figure 2A, the correlation between the % BF at the time of OVX and weight gained from 8.5 to 9.5 weeks of age was poor (*r* = 0.43). This improved slightly when change in body weight was measured over 2 weeks (8.5–10.5 weeks of age, *r* = 0.59, data not shown) and was further improved when extended to 8 weeks (8.5–18.5 weeks of age, *r* = 0.69, data not shown). However, these changes in body weight were still not as predictive of adult adiposity as the <1 week weight gain was in numerous studies in the males described above. Thus, we investigated other potential measures that could be used to accurately screen for the OP and OR phenotypes in females. Change in % BF over the various time points were measured, but these correlations were no better than changes in body weight (*r* = 0.35–0.63, depending on the cohort of animals and time points measured). However, % BF at 14 weeks of age was highly correlated with adult adiposity (Figure 2B; *r* = 0.70–0.81, depending on the cohort). These correlations appeared to be stronger when controlling for the age at the time OVX body composition was analyzed (*r* = 0.79–0.84). At 18 weeks of age, the correlation strengthened even further (*r* = 0.89). However, in our opinion, the added time and cost associated with the additional month of animal housing to delay the separation is likely not warranted. Importantly, our data indicate that % BF early in life (9 weeks of age in this study) is not an accurate predictor of % BF at maturity (*r* = 0.54), and approximately one-third of rats would have been incorrectly categorized (OR, mid, OP) if this early marker of body composition was used. Additional studies will be required to further determine if a time point between 9 and 14 weeks is predictive of long-term adiposity. In the meantime, our approach is to refrain from screening until ~14 weeks of age to identify the OR and OP phenotypes in females.

## Lessons Learned from OP/OR Rats

### Preexisting Differences That May Contribute to Their Predisposition for Obesity

As previously mentioned, one limitation of an OP/OR model that requires stratification based on weight gain is that it is difficult to assess differences between OP and OR phenotypes prior to the dietary challenge. However, an inherent assumption that has been repeatedly validated by our group is that once classified as either OP or OR, rats remain within their respective groups for the remainder of the experimental protocol, suggesting that there are likely preexisting differences between these animals even before the obesogenic challenge. Although our data on differences in OP/OR rats prior to a HF diet challenge are limited, one study did examine preexisting differences in skeletal muscle, and it was observed that OR rats had a significantly higher proportion of type I muscle fibers in the medial head of the gastrocnemius muscle than OP rats (muscle biopsy), suggesting that differences in muscle fiber composition may play a role in determining susceptibility to diet-induced obesity (22).

Additional knowledge in this field comes from studies by Levin et al. Initially, they identified Sprague-Dawley rats as being prone to become DIO or DR on the basis of high vs. low 24-h urine norepinephrine (NE) output (26). Using this approach, DIO-prone rats were observed to have significant reductions in heart, pancreas, and hypothalamic NE turnover, potentially indicating that differences in NE metabolism may be involved in the development of DIO on high energy diets (41). DIO-prone rats also exhibited greater arcuate nucleus NPY mRNA expression, fewer arcuate nucleus projections, leptin resistance, and abnormalities in serotonin turnover compared to DR-prone rats under pre-obese chow-fed conditions (42–45). Similarly, leptin receptor mRNA expression (46), counter regulatory responses to insulin-induced hypoglycemia (47), and central insulin signaling (48) have all been observed to be lower in the inbred line of DIO rats compared to DR rats. Collectively, these observations are indicative of abnormalities in hypothalamic pathways involved in energy homeostasis, all of which may contribute to the development of DIO when presented with a high energy diet.

While the research focus of Levin’s group has primarily been the brain, we have directed our efforts toward differences in whole body and the periphery. Briefly, we have observed that regardless of gender, LF diet fed inbred lines of DIO and DR rats do not differ with respect to total energy expenditure (TEE), food intake, or physical activity (49). Others have also observed no differences in physical activity across the DIO and DR phenotypes on chow diet (50). Interestingly, despite similar intakes and energy expenditure on the LF diet, a greater rate of lipid disappearance was observed in the DR rats compared to DIO rats, suggesting greater basal lipid oxidation in DR rats (49).

### Differential Response to an Obesogenic Diet

In general, following provision of a HF diet, both OP and OR rats initially experience a positive energy imbalance. The OR rats, however, appear to sense the nutrient overload and adjust their food intake and increase their energy expenditure to reestablish energy balance. In doing so, OR rats exhibit an increase in the oxidation of dietary fat. In contrast, OP rodents continue to eat to excess until expenditure increases from their accumulated mass to reestablish energy balance. Although OP rats have a markedly higher food intake, greater intake explains some, but not all of the variance in body weight gain between the OP and OR phenotypes (18, 51). These differences in food intake suggest that there are preexisting differences in regions of the brain that regulate feeding behavior. The findings from Levin’s group with respect to these neuronal differences are beyond the scope of this review; however, we will briefly summarize some of their key findings. One week of HF feeding results in OP rats having elevated leptin, insulin, triglycerides, and glucose, along with increased lipoprotein lipase activity (LPL) in adipose tissue and galanin expression in the paraventricular nucleus (30, 49, 51). It is also noteworthy that OP rats have lower skeletal muscle LPL activity and a decline in the ratio of beta-hydroxyacyl-CoA dehydrogenase to citrate synthase activity, indicating a rapid decline in the capacity for lipid transport and the muscle to metabolize lipids (30, 52). OP rats are also characterized by a preferential trafficking of dietary lipid to adipose tissue for storage, whereas OR rats have greater trafficking of dietary lipid to skeletal muscle after 1 week of HF feeding (52). After 4–5 weeks of HF feeding, OP rats continue to consume more than OR rats, have a higher 24-h respiratory quotient (RQ) (indicating lower relative fat oxidation), and higher plasma levels of free fatty acids (FFA) (18). Insulin sensitivity is also lower in OP rats, which is the result of both lower glucose uptake and lower glucose disposal in skeletal muscle (18, 23). Although we have observed no differences in spontaneous physical activity (SPA) following 1 week of HF feeding (49), others have shown that DIO rats have lower SPA after both 4 and 10 weeks on a HF diet, findings that have been linked to the function or orexin signaling (50, 53, 54).

### Established Obesity – Differences between DIO and DR Rats

Once obesity has been established and the rate of weight gain declines, there are concomitant reductions in the differences in RQ and the measured energy imbalance between DIO and DR rats (51). Coincident, or possibly the cause of the normalization of energy and nutrient balances, is the finding that many hypothalamic differences/abnormalities are also normalized between DIO and DR rats (44). Regardless, DIO rats generated from the aforementioned screening protocol exhibit many of the metabolic derangements and hallmarks that are linked to obesity in humans, including insulin resistance, glucose intolerance, lower spontaneous physically activity, and impaired fat oxidation (40, 49, 52, 55, 56).

### DIO/DR Differences Unique to Females

**Figure 3:**
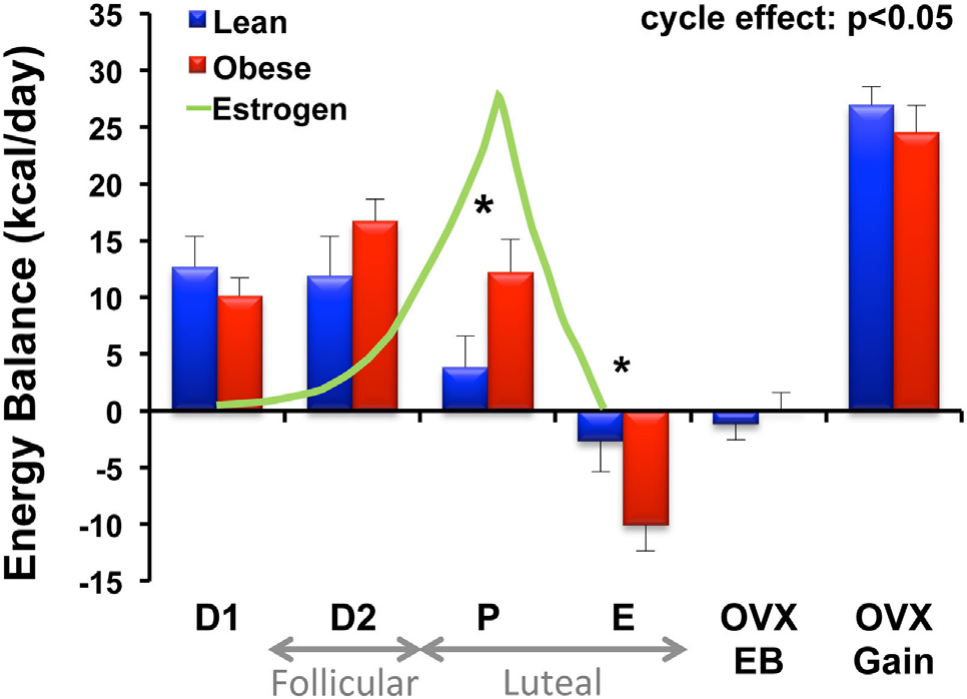
**Energy balance across the estrous cycle and following OVX in lean and obese rats**. Energy balance (intake – expenditure) was measured in lean and obese rats during each phase of the estrous cycle [diestrus 1 (D1), diestrus 2 (D2), proestrus (P), and estrus (E)], immediately following surgical ovariectomy (OVX) while in energy balance (OVX-EB), and during OVX-induced rapid weight gain (OVX-Gain). Relative circulating estrogen levels across the cycle (D1–E) and corresponding follicular and luteal phases of the human menstrual cycle are indicated. Obese rats experience greater fluctuations in energy balance across the cycle compared to lean rats. A rise in circulating estrogens is associated with a decrease in food intake (and energy balance) in the lean; however, this response is delayed in the obese. *Significant difference between lean and obese groups (*P* < 0.05). Modified from Ref. (40).

Aside from the initial studies that showed no difference in EI, TEE, or activity levels between DIO and DR rats, our work with the female-specific aspect of this model has not specifically addressed the differences between male and female rats in terms of their propensity to become obese. We have, however, performed comprehensive metabolic studies of the mature lean and obese animals across the estrous cycle, and during the initial stages of weight gain following surgical OVX (40). We observed that obese animals experienced greater fluctuations in energy balance across the 4-day estrous cycle than their lean counterparts, and this was driven by greater variability in food intake across the cycle (Figure 3). A rise in estrogen levels during the proestrus phase of the cycle underlies a reduction in food intake, and this estrogen-mediated response appears to be delayed in obese animals (Figure 3). While circulating estradiol levels were not significantly different between the DIO and DR animals, we suspect that the inherent impairment in leptin and/or insulin sensitivity in the obese may impart reduced sensitivity to the effects of estrogen at this stage of their cycle. Additional studies are needed to examine this possibility. We further found that cycling obese rats were less active, expended more energy per movement, and oxidized more carbohydrate than lean rats. Despite these phenotypic differences across the cycle, OVX induced a large positive energy imbalance in both obese and lean rats, which resulted primarily from an increase in energy intake in both groups. TEE was not altered in either group, despite the fact that they were eating more food. Our interpretation of these observations is that the increased thermic effect of food (from the greater food intake) is essentially balanced out by any reduction in the non-resting energy expenditure (NREE) that occurs from the decline in physical activity levels.

### Characterizing the Metabolic Propensity to Regain Weight after Weight Loss

**Figure 4:**
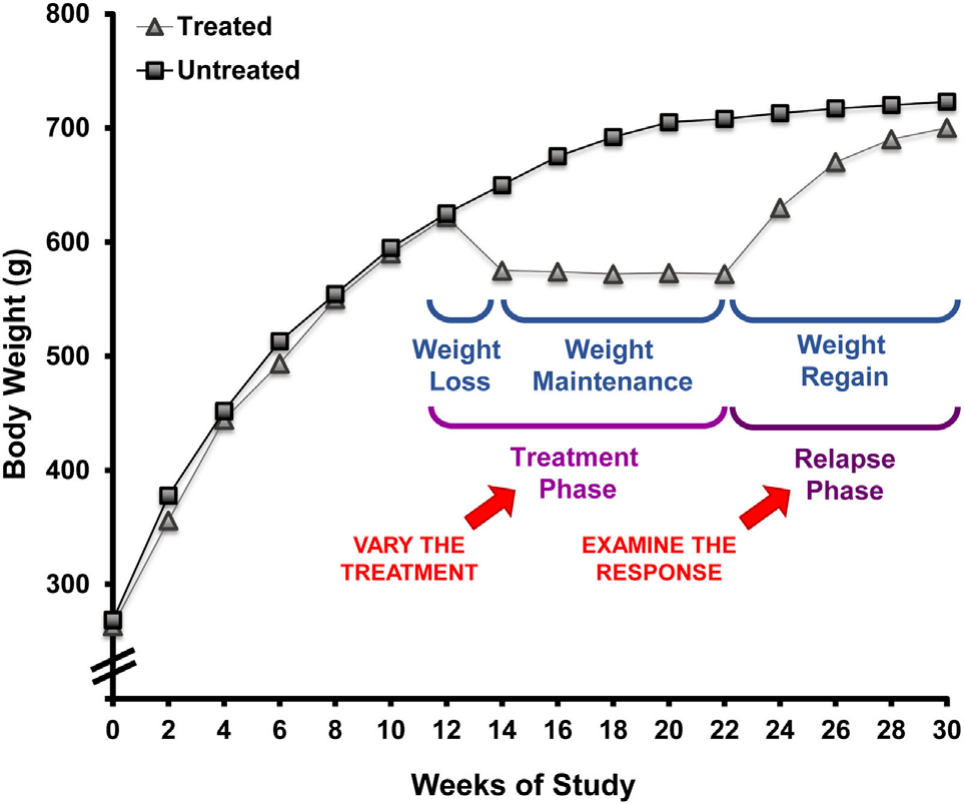
**Rodent paradigm to study the metabolic propensity to regain weight after weight loss**. This paradigm, employing obesity-prone rats, has well-defined primary and secondary outcomes that describe the metabolic propensity to regain weight after prolonged weight reduction. This approach can be applied to test weight reduction strategies for their ability to overcome the metabolic pressures driving weight regain by modifying the environmental conditions in the treatment phase and examining the response during the relapse phase.

Over the past decade, we have employed the OR/OP model to study the phenomenon of weight regain after weight loss. The paradigm we developed to model the human condition is shown in Figure 4. Following the standardized screen to identify the OR and OP phenotypes described above, the young rats are maintained in the obesogenic environment (HF diet; limited physical activity) for 16 weeks, during which excessive weight gain occurs in what would be equivalent to childhood and adolescence. As the rats mature, growth rates slow, the gain in body weight and fat-free mass plateaus, and further weight gain comes slowly and primarily in the form of fat mass. The rats are then given a two-step treatment regimen (weight loss followed by weight maintenance) that reflects the most common approach used in humans: restricted consumption of an LF diet. The rats are fed a calorie-restricted LF diet that induces a 10–15% loss in weight that is primarily fat mass. A LF provision, adjusted on a daily basis, is given so that weight is maintained at this reduced level. In some studies, weight reduction has been sustained with intake-regulated maintenance for up to 16 weeks, a period of time that would reflect several years of weight reduction in humans (57). The propensity to regain weight is then characterized by allowing the rats to have free access to a specific diet while monitoring body weight, body composition, and pertinent aspects of metabolism as they relapse to the obese state. Our assertion is that this paradigm reflects the human condition with respect to:

(1) Genetic pressures – polygenic predisposition to become obese under obesogenic conditions;
(2) Obesogenic conditions during formative development – maturation in an obesogenic environment;
(3) The most common approach to weight reduction – restricted/controlled intake of an LF diet; and
(4) The most common failure – not controlling intake.

In our first set of studies, we examined weight regain immediately after weight reduction and assessed how energy balance and fuel utilization were altered with prolonged weight reduction. As expected, the drive to regain could be described by an increased rate of gain and a return to the previous level of body weight and fat mass. We observed that prolonged (8-week) weight reduction was accompanied by a persistent reduction in TEE that was due in part to a sustained reduction in resting energy expenditure (REE). By adjusting for the variability attributed to variations in fat-free mass, we observed an enhancement in metabolic efficiency, meaning the suppression in resting metabolic rate was greater than what would be predicted based the decreased mass of metabolically active tissues that occurred with weight reduction. Like adult humans that are obese, fluctuations in weight were primarily due to changes in fat mass rather than changes in fat-free mass. Furthermore, relapsing rats had a tendency to burn carbohydrate rather than fat. Importantly, this study established the weight loss/weight regain paradigm, describing the basic approach and technical tools used for its application (34).

### The Role of Timing in the Weight Regain Process

#### Length of Time in Weight Maintenance

**Figure 5:**
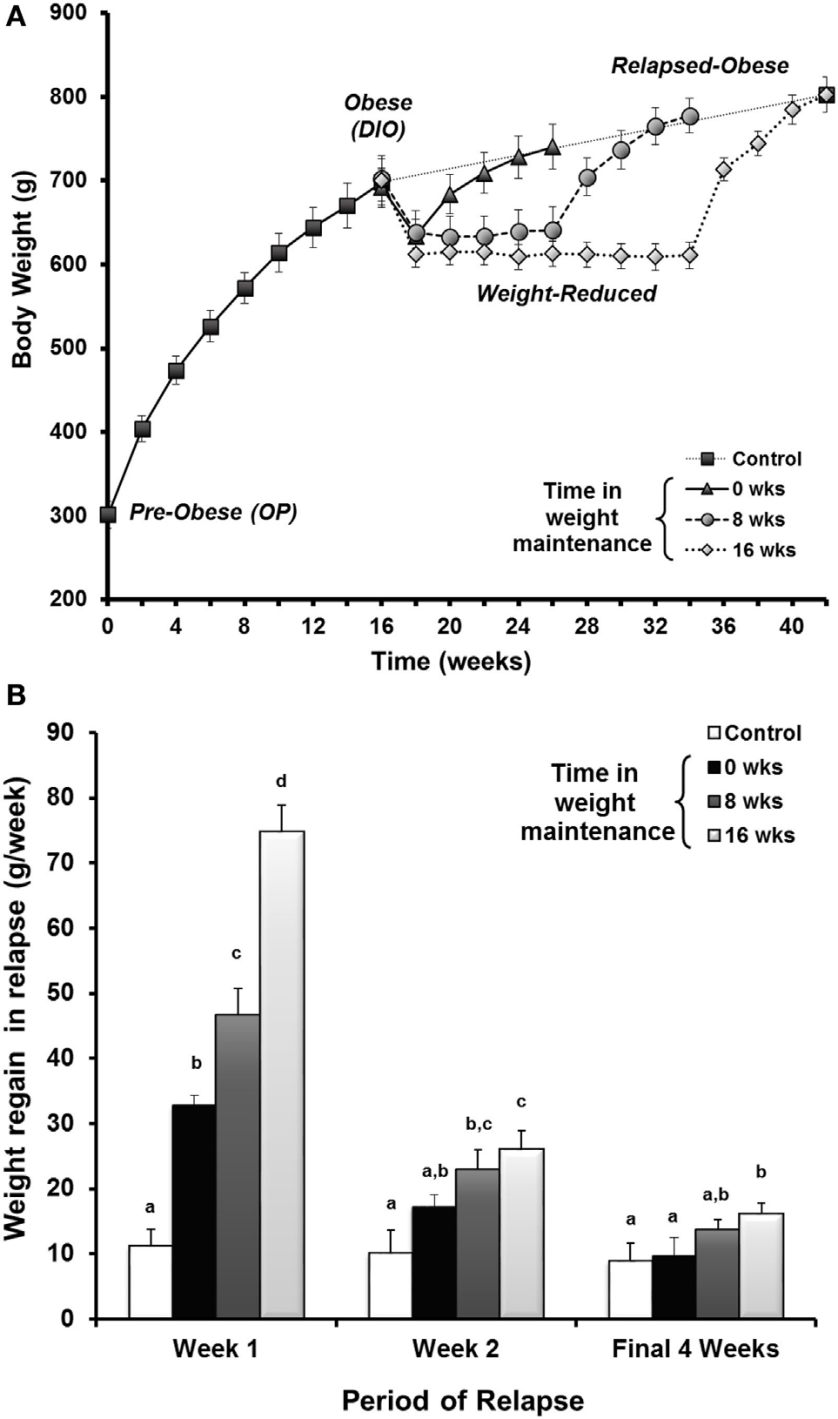
**Drive to regain weight increases with time and is higher early in the relapse period**. **(A)** Rats in the paradigm examining the length of time in weight maintenance vs. the drive to regain weight. Obesity-prone (OP) rats were maintained on a HF diet for 16 weeks to induce obesity. Rats were calorically restricted on an LF diet to induce 10–15% loss, maintained at this reduced weight for 0, 8, or 16 weeks, then allowed to relapse with free access to LF diet. **(B)** The rate of weight regain shown by time in maintenance at various times during the relapse period. The rate of weight regain for relapsed-obese rats is represented as the average for the first week, the second week, and the final 4 weeks of the relapse period. Data are expressed as means ± SE. With each time period, groups with the same letter designation are not significantly different. Modified from Ref. (34).

Based upon observations from the National Weight Control Registry, the paradigm was applied to address whether increasing the time of weight maintenance would attenuate the metabolic drive to regain weight. Given the sustained reduction in TEE with prolonged weight reduction in our prospective study, we were not overly optimistic that this would be the case. To better understand the aspects of metabolism promoting regain, we performed a large cross sectional study in male rats examining the weight reduced state at 0, 8, and 16 weeks of weight maintenance, before and after 8 weeks of relapse (34). The time in weight maintenance was equivocal to a weight-reduced human keeping the weight off for ~10 years (57). A portion of these data is shown in Figure 5. We observed that the rate of regain increased with time in weight maintenance (Figure 5A), but the level of defended body weight and adiposity was drifting higher (obese rats switched to LF diet, dotted line). The animals were defending a target weight that was drifting upwards while they were weight reduced, an effect we attribute to age. Regardless, the increased rate of regain indicated that the metabolic pressures driving regain were greater as the time in weight maintenance increased. This effect on the rate of regain was most profound in the first week of the relapse period (Figure 5B). In addition, we observed that the decreased TEE and enhanced metabolic efficiency observed in our previous study remained unchanged when the length of time in weight maintenance increased. These metabolic adaptations that are thought to be contributing to the biological drive to regain weight did not resolve, even with long-term weight loss maintenance. While some similar mechanisms are likely to underlie the biological drive to regain weight in females, we know that there are many sex-specific differences that also likely exist. Our lab is currently performing these same studies in females to fully characterize the nature and extent of these differences, particularly with respect to (a) the extent and kinetics of the weight regain process and (b) the effects of menopause/loss of ovarian hormones on this process.

#### Prospective Analysis of *Early* Relapse

**Figure 6:**
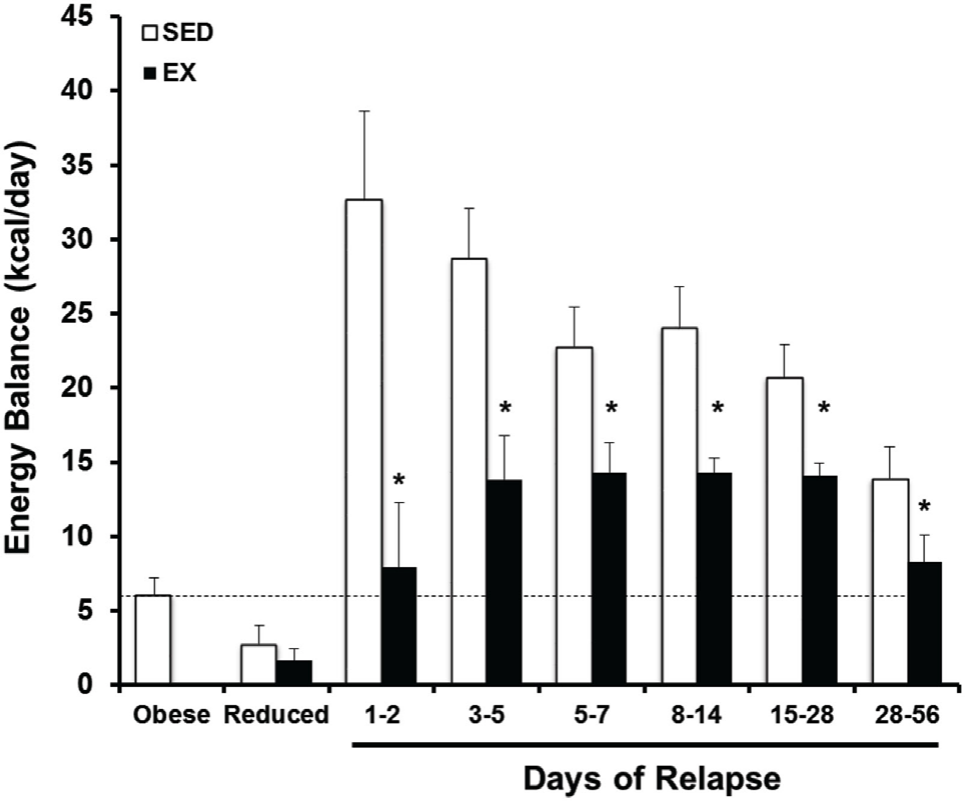
**Prospective analysis of the energy gap during weight regain with and without exercise**. Energy balance [energy intake – total energy expenditure (EI-TEE)] is shown for obese, weight reduced, and relapsing rats either with (EX) or without (SED) treadmill exercise for several time periods during relapse. During weight regain, the energy gap (energy imbalance) resolves gradually as body weight is gained. Further, exercise reduces the energy gap both by suppressing EI and increasing TEE. *Significant difference between SED and EX rats during that time period, *P* < 0.05. Modified from Ref. (55).

To examine this critical early period, energy balance, fuel utilization, and regain were monitored prospectively through the first 2 weeks of relapse. During this time, almost half of the lost weight had been regained (34). We observed that both an increase in drive to eat and a decrease in expenditure contributed to the large energy gap, and neither side of the energy balance equation had normalized by the end of 2 weeks. Enhanced metabolic efficiency persisted throughout this early period of relapse and contributed to the suppressed TEE. In other studies, we utilized nutrient tracers in combination with metabolic phenotyping to examine fuel trafficking during the early stages of weight regain (58). During weight regain, we observed that the oxidation of dietary fat was substantially suppressed and that ingested fat was preferentially trafficked to adipose tissue for storage. Accompanying this shift in metabolism early in relapse was an increased number of small adipocytes, which would presumably provide an ideal receptacle for the excess ingested energy. When taken together, observation of DIO rats in this paradigm of weight regain suggest that adaptive changes in muscle and adipose tissue establish a metabolic context for rapid, energetically efficient weight regain. In subsequent studies, we have observed how regular exercise counters this metabolic drive to regain weight early in relapse; exercise decreases the energy imbalance or energy gap (Figure 6) both by reducing appetite and by increasing the level of expended energy during weight regain (55). Using nutrient tracers, we provided evidence suggesting that regular exercise increases the oxidation of dietary fat and traffics excess energy through more expensive pathways of deposition (59, 60). We have examined the tissue-specific mechanisms of these beneficial effects of exercise in both skeletal muscle (59) and adipose (60), and our analysis of the effects in liver will be forthcoming.

#### Prospective Analysis of *Complete* Relapse

**Figure 7:**
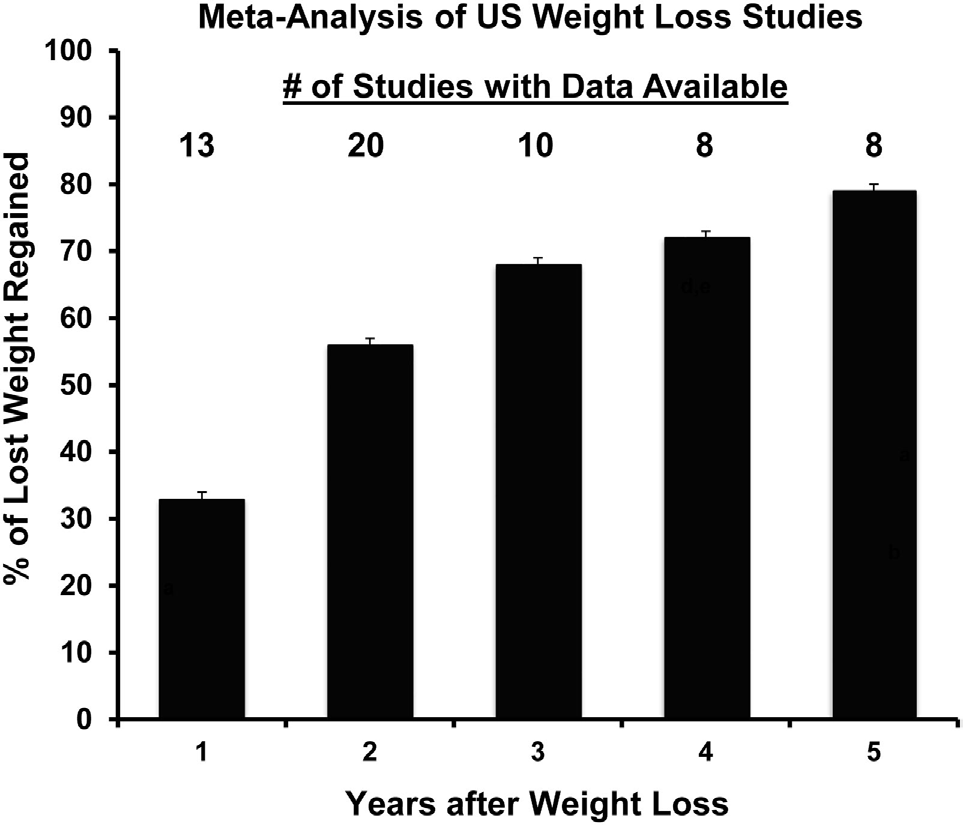
**Propensity to regain weight following weight loss: summary of data from a meta-analysis of US weight loss studies**. The percentage of lost weight that is regained over a 5-year period according to a meta-analysis of 29 human weight loss studies. Adapted from Ref. (61).

Our studies of DIO rats in this weight regain paradigm have also examined the later stages of the relapse process to provide a more complete biological picture of weight regain after weight loss. We were interested in following the resolution of the energy gap, non-protein RQ, fuel utilization, and the energetic efficiency of weight regain. The pattern of regain in our rat paradigm reflected the pattern of regain in a meta-analysis of a large number of human regain studies (61), which some have suggested reflects a first-order relationship in the resolution of this biological drive (Figure 7). Our observations extended our previous findings by showing that the enhanced metabolic efficiency and suppressed TEE persist throughout the process of relapse. Both increased intake and suppressed expenditure led to a large energy imbalance, or energy gap, which resolves gradually as the weight returns (Figure 7). While intake returns to levels observed before weight loss, TEE and REE never completely resolved even after the rats had returned to their previous weight. Both the reduction in feed efficiency and the elevation in non-protein RQ declined after week 2, suggesting that this efficient weight gain and shift in fuel use was most profound early in relapse when much of the lost weight returns (56). As expected, we and others have observed, at least in males, that exercise and physical activity attenuates the biological drive to regain weight early in relapse and leads to a lower body weight and fat mass (55, 59, 60). These studies support the notion that in males, exercise attenuates the drive to eat and increases expended energy above and beyond the additional energetic cost of the exercise bout, and that these effects persist throughout the entire relapse process. However, we have also observed that these beneficial effects of exercise may be greatly diminished if weight regain occurs on an obesogenic diet (62).

#### Use of DIO/DR Rats for Studying Obesity Therapeutics

To date, we have used the DIO/DR rats to develop a broad picture of the biological drive to regain weight after weight loss, and we have used the experimental paradigm of weight regain in DIO rats to examine the impact of one of the most effective strategies for weight loss maintenance: regular physical activity. Given the importance of the biological adaptations in driving the weight regain process (2), we assert that the use of this model in this paradigm may provide valuable information about specific therapeutic strategies or combinations of strategies that are designed to attenuate the biological drive to regain weight after weight loss. In addition, our data suggest that it is critical to assess the sex-specific differences in the biological drive to regain weight, as well as the sex-specific differences in the efficacy of therapeutics and strategies targeting these biological adaptations for weight loss maintenance. The development of a more effective screening process for females discussed earlier will greatly facilitate this important work moving forward.

### DIO/DR Studies in Females

#### Obesity and the Loss of Ovarian Function

As previously stated, we have applied the DIO/DR model to study the effects of obesity on energy homeostasis in females (40). By merging the DIO/DR model with other common approaches used in preclinical research, we have extended the utility of the DIO model for studies of menopause and breast cancer. Menopause is a very complex transition accompanied by a wide array of metabolic derangements in numerous tissues of the body. While there are several groups that have performed elaborate clinical studies either across the natural menopause transition (63) or in studies of ovarian hormone suppression (64–66), it is generally difficult to study the menopause transition in women because of the wide variations in the length of time for this transition, the variable age of onset, and the fact that it is generally only identified retrospectively. To overcome these logistical challenges in preclinical studies, we have utilized surgical OVX to mimic the loss of ovarian function (38, 40, 67, 68). This approach reflects some of the metabolic consequences of the menopausal transition, but it has the added advantage of a clear demarcation of the loss of ovarian hormone production for precise timing for follow-up analyses. We have specifically merged the DIO/DR model with this surgical intervention to study the impact of preexisting obesity on the loss of ovarian hormone production. Following OVX, we have shown that both DIO (obese) and DR (lean) rats exhibit a 3- to 4-week period of rapid weight gain that is accompanied by increased energy intake and reduced SPA levels (38, 40). While DIO and DR rats generally gain the same amount of weight, the weight gain appears to be somewhat slower or delayed in the DIO females (38). While activity levels are reduced after OVX in our model, our data suggest that in this paradigm, weight gain is primarily a result of increased food intake, rather than changes in energy expenditure (40).

#### Obesity and Postmenopausal Breast Cancer

One of the many comorbidities associated with overweight and obesity is an increased risk for, and mortality from, many cancers including breast cancer (69–72). Surprisingly, obesity’s impact on breast cancer prior to menopause is relatively modest and in some cases has even been shown to be protective (73). After menopause, however, obesity increases the incidence, progression, and eventual mortality from breast cancer by up to 40% compared to women at a healthy weight (74). The risk is highest in women with a history of weight gain throughout life, suggesting that a crisis in obesity-driven breast cancer is likely to occur with the current generation of youth, where obesity rates are approaching an unprecedented 20% (75). Despite the known link between obesity and postmenopausal breast cancer, the mechanisms underlying this association are not fully understood. This represents a significant gap in our knowledge, and identifying mechanisms of risk and targets for intervention is critical.

To pursue a deeper understanding of the biological aspects underlying this relationship, we merged the DIO–DR/OVX model with a common preclinical approach to studying mammary tumor biology. Prior to the OP/OR screening, a chemical carcinogen (MNU) is delivered during mammary gland development. The animals are then matured under obesogenic conditions into DIO and DR rats, after which they are subjected to surgical OVX. Merging the models in this fashion has generated an experimental paradigm that can further our understanding of the impact of obesity on postmenopausal breast cancer.

Mammary tumors generated in these animals are reflective of the human condition with respect to the histological characteristics and estrogen receptor (ER) status of the tumors (38, 39). Further reflective of humans, the effect of obesity on mammary tumor incidence is minimal prior to OVX, and the emergence of an obesity effect occurs only after the OVX surgery. Specifically, in response to OVX, obese rats exhibit fewer tumors that regress, more tumors that progress, and more tumors that newly emerge (38). Of all the characteristics of the obese phenotype that were examined, the strongest relationship with tumor promotion was observed with the energetics of weight gain during the ~3- to 4-week period of rapid weight gain that followed OVX. During this time, both groups experienced a dramatic increase in the rate of weight gain. However, despite the two groups eating similar amounts of food, DIO rats gained less weight (*p* < 0.01). Consequently, feed efficiency during this transient period of rapid gain was lower in DIO rats (*p* < 0.001). Feed efficiency during this 3-week period of rapid weight gain was inversely associated with the change in tumor multiplicity (*r* = −0.64, *p* < 0.001) and burden (*r* = −0.60, *p* < 0.001) over the entire post-OVX period. Rats that experienced a lower rate of weight gain and a lower efficiency of storing ingested fuels during this distinct time period after OVX also had a higher level of tumor progression. These observations suggest that the impact of OVX on energy balance and fuel utilization is different for DIO and DR rats in this paradigm and that this difference may affect the latency, survival, and growth of mammary tumors.

#### OVX-Induced Overfeeding – An Example of Metabolic Inflexibility

We subsequently performed a nutrient tracer study in DIO/DR rats during the early stages of OVX-induced weight gain. Tumor bearing DIO and DR rats were studied after OVX both while in energy balance, and while experiencing their natural OVX-induced positive energy imbalance (and subsequent weight gain) (39). These studies were performed in a metabolic phenotyping system in which energy intake, expenditure, and tissue-specific nutrient trafficking could be carefully measured. In DR rats, the OVX-induced energy imbalance was accompanied by a higher level of glucose uptake (^3^H-2-deoxyglucose) in the mammary gland adipose depot, and similar trends were seen in the liver, retroperitoneal adipose, and skeletal muscle. Overfeeding in this context had no effect on the glucose uptake in the tumors of DR rats. Our observations in DIO rats were in direct contrast to those in DR rats. Glucose uptake was unaffected by the OVX-induced positive energy imbalance in all tissues of the DIO rats, with the exception of the tumors, where glucose uptake was increased. Changes in whole body fuel utilization (RER) and dietary fat oxidation in response to this caloric excess also tended to be blunted in the obese. Taken together, our studies of this critical period of OVX-induced overfeeding indicate that DIO rats have an inability to clear and store nutrients in peripheral tissues, but excess nutrients are readily taken-up by mammary tumors.

We have interpreted these observations from the perspective of metabolic flexibility, which we broadly define as the ability to change or adjust nutrient metabolism in response to a metabolic challenge. The challenge in this context is OVX-induced overfeeding. In the insulin-resistant DIO rats, their peripheral tissues exhibit a blunted or impaired response to the excess energy, while their tumors readily take up the excess energy. In the DR rats, their peripheral tissues exhibit greater flexibility in their response to the challenge and are more capable of clearing and metabolizing the excess energy. We would assert that this impaired ability to respond to metabolic stress may underlie many of the metabolic derangements and accompanying pathologies associated with obesity. Even so, metabolic flexibility is a concept that is difficult to specifically define and even harder to study. The DIO/DR model may prove a useful tool to study this important concept in well-defined metabolic contexts.

## Conclusion

Over the past two decades, the impact of obesity on overall health and wellness has emerged as a major crisis. The study of OP/OR and the DIO/DR rat model has proven to be a valuable tool in translating basic science studies of energy balance and body weight regulation to the human condition and in furthering our understanding of the physiologically relevant condition of obesity. The strengths of this approach are the polygenic nature of the adipose disposition, and that it has been shown to reflect the human condition in a number of ways. The Levin model of inbred lines has been particularly useful in that they have been used to study OP and OR phenotypes prior to and during the development of a particular adiposity phenotype. While the commercial availability of Levin’s valuable model continues to be questionable, researchers may continue to pursue the OP/OR selection of outbred strains to produce the DIO/DR phenotypes to further our understanding of obesity and its metabolic complications. Both the inbred and outbred strains have proven extremely valuable for obesity studies of female physiology and have elucidated critical sex-specific differences of obesity and its metabolic complications.

## Author Contributions

All the authors contributed to the writing of this review, and they have all read and approved the final manuscript.

## Conflict of Interest Statement

The authors declare that the research was conducted in the absence of any commercial or financial relationships that could be construed as a potential conflict of interest. The reviewer AB and handling Editor declared their shared affiliation, and the handling Editor states that the process nevertheless met the standards of a fair and objective review.
